# Quality indicators for multiple sclerosis

**DOI:** 10.1177/1352458510372394

**Published:** 2010-08

**Authors:** Eric M Cheng, Carolyn J Crandall, Christopher T Bever, Barbara Giesser, Jodie K Haselkorn, Ron D Hays, Paul Shekelle, Barbara G Vickrey

**Affiliations:** 1 Department of Neurology, David Geffen School of Medicine, University of California, Los Angeles, CA, USA.; 2 Department of Neurology, VA Greater Los Angeles Health Care System, Los Angeles, CA, USA.; 3 Department of Medicine, David Geffen School of Medicine, University of California, Los Angeles, CA, USA.; 4 Multiple Sclerosis Center of Excellence-East, Research and Neurology Services, VA Maryland Health Care System, Baltimore, MD, USA.; 5 Department of Neurology, University of Maryland School of Medicine, Baltimore, MD, USA.; 6 Multiple Sclerosis Center of Excellence-West, VA Puget Sound Health Care System, Seattle, WA, USA.; 7 Department of Rehabilitation Medicine, University of Washington School of Medicine, Seattle, WA, USA.; 8 Departments of Epidemiology, University of Washington School of Medicine, Seattle, WA, USA.; 9 Department of Medicine, VA Greater Los Angeles Health Care System, Los Angeles, CA, USA.

**Keywords:** health services research, multiple sclerosis, outcome research, quality indicators

## Abstract

Determining whether persons with multiple sclerosis (MS) receive appropriate, comprehensive healthcare requires tools for measuring quality. The objective of this study was to develop quality indicators for the care of persons with MS. We used a modified version of the RAND/UCLA Appropriateness Method in a two-stage process to identify relevant MS care domains and to assess the validity of indicators within high-ranking care domains. Based on a literature review, interviews with persons with MS, and discussions with MS providers, 25 MS symptom domains and 14 general health domains of MS care were identified. A multidisciplinary panel of 15 stakeholders of MS care, including 4 persons with MS, rated these 39 domains in a two-round modified Delphi process. The research team performed an expanded literature review for 26 highly ranked domains to draft 86 MS care indicators. Through another two-round modified Delphi process, a second panel of 18 stakeholders rated these indicators using a nine-point response scale. Indicators with a median rating in the highest tertile were considered valid. Among the most highly rated MS care domains were appropriateness and timeliness of the diagnostic work-up, bladder dysfunction, cognition dysfunction, depression, disease-modifying agent usage, fatigue, integration of care, and spasticity. Of the 86 preliminary indicators, 76 were rated highly enough to meet predetermined thresholds for validity. Following a widely accepted methodology, we developed a comprehensive set of quality indicators for MS care that can be used to assess quality of care and guide the design of interventions to improve care among persons with MS.

## Introduction

Multiple sclerosis (MS) is a neurological disorder that affects 400,000 people in the United States.^1^ Gaps in care quality exist for many chronic diseases^2^^,^^3^ and have been reported for aspects of MS care.^4^ However, gaps in many other aspects of MS care have not been studied. Identifying gaps in care quality requires tools for measuring the quality of comprehensive MS care. Understanding why gaps in care quality exist is fundamental to designing healthcare delivery system interventions.^5^^,^^6^

The quality of medical care can be measured through medical care processes or patient outcomes.^7^ While traditional MS measures such as the Expanded Disability Status Scale (EDSS) scores are appropriate for assessing outcomes of participants enrolled in randomized controlled trials (RCTs), they are less useful outside of such settings because differences in outcomes may be attributable to factors other than the quality of medical care delivered.

As an alternative to patient outcomes, major stakeholders in healthcare have developed and used quality indicators to measure processes of care.^8^^,^^9^ A scientifically rigorous methodological approach called the RAND/UCLA Appropriateness Method (RAM) is a widely utilized technique for developing indicators to measure processes of care in many conditions, including neurological conditions such as stroke,^10^^,^^11^ Parkinson’s disease,^12^ dementia,^13^ and epilepsy.^14^ The goal of RAM is to identify processes of care to which adherence is strongly associated with better health outcomes.

We applied RAM to develop a comprehensive set of quality indicators to measure the quality of healthcare of persons with MS.

## Materials and methods

### Overview of the stages and techniques pursued in the research study

**Figure 1.**
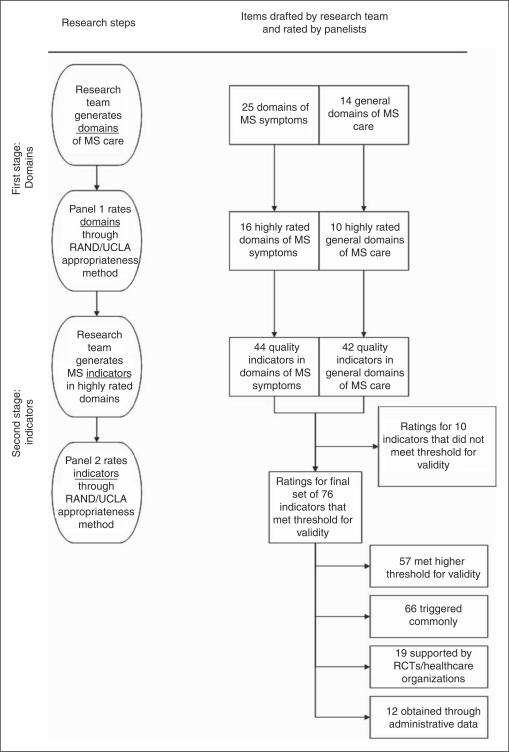
Flow diagram of items drafted by research team and then rated by the two panels.

We used a modified version of the RAM in a two-stage process to (1) identify relevant MS care domains and then (2) draft indicators and rate their validity (Figure 1). Because MS is characterized by a wide spectrum of symptoms and available disease-modifying and symptom-targeted treatments,^15^^–^^17^ there is a vast number of potential quality indicators that could be drafted for MS care. By first identifying the most important domains for MS care, the research team could then prioritize a resource-intensive literature review to identify candidate indicators.

An overview of the RAM is presented here. RAM is a systematic method of combining evidence with expert judgment and contains characteristics of both the Delphi method and nominal group techniques.^18^^–^^20^ First, a research team performs a comprehensive review of the literature. Based on the literature review, the research team drafts a set of items to be rated, and mails these items to panelists to be rated in private without consulting one another. Panelists then mail their ratings back to the research team. A face-to-face meeting of the panelists is then convened to review the de-identified ratings, discuss reasons for disagreement in ratings, and anonymously re-rate the items. Finally, the research team applies pre-determined statistical thresholds of the ratings to identify items of high importance.

### Assembly of an expert panel of nationally recognized MS stakeholders

We identified 17 general health and MS-specific organizations that comprehensively represent stakeholders of MS care (see the list in the acknowledgements) and obtained from each organization a list of nominees who could serve on a panel to rate MS care domains. We selected nominees to attain a diverse range of clinical disciplines and geographical locations. We invited our first-choice nominees to participate, and they all accepted, and we refer to this group as Panel 1. Panelists were not told which organization nominated them and were instructed to rate items based on their own perspective and not from the perspective of any organizations to which they are affiliated. The multidisciplinary panel comprised major stakeholders of MS care including four persons with MS, directors of MS patient advocacy organizations, neurologists, rehabilitation physicians, nurses, therapists, and healthcare administrators.

## First stage

### Generating a comprehensive set of MS care domains

We used three sources of data to inform development of a comprehensive set of MS care domains. First, we interviewed a convenience sample of 10 persons with MS across different mobility stages receiving care at the VA Greater Los Angeles (VA GLA) or University of California, Los Angeles (UCLA) to understand their perspectives on living with MS. A semi-structured interview tool that assessed demographics, MS symptoms, physical functioning, emotional well-being, social functioning, current MS symptoms and care, and outlook for the future was used during these sessions. All interviews were audiotaped, and summaries of each interview were shared with the research team.

Next, the research team performed a systematic review of PubMed using Medical Subject Headings terminology, and then performed reference mining of relevant studies. We also reviewed the websites of the National Guideline Clearinghouse,^21^ Cochrane Database of Systematic Reviews,^22^ United States Preventive Services Task Force (USPSTF),^23^ American Academy of Neurology,^24^ and the National Multiple Sclerosis Society^25^ for guidelines, indicators, reviews, and large trials providing or summarizing scientific evidence relevant to MS care. The International Classification of Functioning, Disability and Health established by the World Health Organization was used to organize an initial set of 70 MS care domains.^26^ The research team deleted domains that were not well supported by the literature review and combined others to reduce redundancy. Individual phone calls with panelists were arranged to obtain feedback on revising the list of MS care domains. A final set of 39 MS care domains were mailed to panelists, including 25 MS symptoms in at least one of four mobility stages of disease: ambulatory without assistance, ambulatory with assistance, wheelchair user, and bed-bound as well as a list of 14 general health domains that are applicable across mobility stages.

### Rating MS care domains

Each panelist was mailed a booklet for rating the MS care domains and a monograph summarizing the literature review. First, panelists were instructed to sort an equal number of MS symptoms within a mobility stage of disease into three tiers of order of importance: highest level of importance, second highest level of importance, and third highest level of importance. Second, panelists were instructed to sort general health domains into three tiers of order of importance. Third, panelists designated three general health domains as indispensable to MS care.

The second round of ratings occurred during a subsequent face-to-face meeting of the panel. Panelists were given their own unique summary rating sheets that contained the de-identified initial distribution of ratings by the entire panel, as well as a reminder of that particular panelist’s own ratings. Thus, panelists could determine how their own ratings compared with the distribution of the entire panel’s ratings, but they could not determine the ratings of any other particular panelist. The members of the research team moderated the discussion to limit the role of any dominant members and encouraged participation from the entire panel. Finally, once discussion of a set of domains was complete, the panelists confidentially re-rated the domains using identical criteria to those used in the first round.

## Second stage

### Generating a comprehensive set of MS quality indicators

The highly rated MS care domains guided a subsequent literature review for drafting quality indicators. Similar of sources used to identify MS care domains were again used to identify potential indicators. Indicators were worded in the form of an ‘IF … THEN …’ or an ‘ALL persons with MS SHOULD…’ statement. An external team of an MS specialist, rehabilitation physician, and an MS nurse not related to the research project reviewed each indicator and suggested further changes to enhance clarity. Ultimately, 88 indicators were drafted across 26 domains of MS care. For Panel 2, several domains were consolidated, reducing the number to 24 domains.

### Rating MS quality indicators

All persons who rated the domains in the first year were invited to participate in the second panel, which we refer to as Panel 2. Because the literature review for indicators in Panel 2 contained more clinically technical information than that for domains in Panel 1, additional clinicians were invited for Panel 2 to ensure there was sufficient expertise to evaluate each indicator. Panel 2 comprised 18 persons, including 4 persons with MS.

A rating booklet and a monograph summarizing the literature supporting each indicator were mailed to the members of Panel 2. Panelists were asked to rate each indicator using a nine-point visual scale of validity, with higher numbers indicating greater validity (see Table 1 for definition of validity and visual scale provided to Panel 2). This definition of validity was adapted from prior RAM studies.^19^^,^^27^ Similar to Panel 1, the research team created personalized feedback sheets for panelists that reminded the panelists of their first round rating and provided the anonymous distribution of ratings of the entire panel for each indicator.

**Table 1.** Definition of the criteria of validity used by Panel 2 to rate MS quality indicators

1. Evidence and opinion supports a link between an indicator and positive MS patient outcomes such as									
• mortality									
• symptoms									
• functional status									
• mental health									
• satisfaction with care, and									
• compliance with evidence-based treatments **AND**									
2. An indicator that applies to a larger proportion of the eligible population will have more impact on the health of the population and thus should have a higher level of validity than an indicator that applies to only a few people, **AND**									
3. An indicator that has a greater impact on the health of an individual person (such as management of phenylketonuria) should have a higher level of validity than an indicator that has a smaller impact on the health of an individual person (such as management of eczema).									
Lowest level of validity					Highest level of validity			
1	2	3	4	5	6	7	8	9
□ Decline to answer									

The second round of ratings occurred during a subsequent face-to-face meeting. Panelists were given the opportunity to suggest changes in phrasing for each indicator. Next, the research team invited discussion of the indicator, particularly when there was lack of consensus in the first round ratings for an indicator. Panelists then discussed the basis for their first round ratings, then confidentially re-rated the indicators.

## Analysis

For the domains of MS symptoms, the one-third of domains with the highest number of panelists rating that domain in the top tier were considered the most important for that stage of disease. For example, of the 22 domains applicable to the MS population who ambulate without assistance, we designated the 8 domains with the highest number of panelists voting them into their top tier as the most highly rated (domains tied for the eighth highest ratings in the top tier were included in the set of most highly rated domains). For the general domains of MS, we included all domains that a panelist identified as indispensable to MS care.

Because the criteria for rating quality indicators used an ordinal scale and the frequencies across the scale values were not normally distributed, indicators were ranked by their median instead of mean ratings. Indicator projects that use a 1–9 rating scale of validity typically accept indicators in the highest tertile of the scale (median ratings of 7, 8, or 9) as valid.^3^^,^^19^^,^^27^ Wilcoxon rank-sum tests were used to compare the ratings between the 4 panelists with MS versus the 14 panelists without MS.

While all indicators that meet thresholds for validity are suitable for measuring quality, measurement programs of healthcare organizations do not have the resources to implement all of them. To provide a basis by which a subset of indicators could be selected, we categorized the final set of valid indicators according to four criteria that may be pertinent to a measurement program. The first criterion is the strength of the panel’s rating, defined as a high median rating on validity (≥8) and narrow dispersion of ratings (≥80% of panelists rated indicator in highest tertile). The second criterion is the frequency with which an indicator was expected to be applicable (defined as applicable to at least 20% of cases within a particular year based on prevalence data identified in the literature review). The third criterion is the level of evidence supporting an indicator (defined as results from an RCT or endorsement by one of the following organizations: the US Food and Drug Administration, the Centers for Disease Control, or the USPSTF). The fourth criterion is the means of measurement, identifying those indicators that could be measured using administrative data.

We obtained approval from the Institutional Review Boards at VA GLA and UCLA to conduct this study. Written informed consent was obtained from all subjects participating in the patient interviews.

## Results

Among the MS-specific domains, bladder dysfunction, cognitive dysfunction, depression, fatigue, and spasticity were highly rated by Panel 1 in at least three of the four mobility stages (Online Table 1). A total of 16 domains fell in the top tier within at least one stage of disease. The 10 general domains of MS care rated highly by Panel 1 are listed in Online Table 2. The general domains that received the most votes by Panel 1 for being indispensable to MS care were ‘At time of diagnosis: Medical evaluation-appropriateness and timeliness’, ‘Disease-modifying agents’, and ‘Establishment, integration, and coordination of care’.

**Table 2.** Abbreviated name of 76 valid indicators

Domain	Abbreviated text of MS indicators that met thresholds for validity
**Domains of MS symptoms**	
*Anxiety*	Management of anxiety
Bladder Dysfunction/ Urinary Tract Infection (UTI)	Assessment of urinary symptoms Assessment for UTI upon hospital admission
	Management of post-void residual urine
	Avoid treatment of asymptomatic bacteriuria
	Test for antibiotic susceptibility with recurrent UTI
	Work-up of chronic subjective bladder symptoms
Bowel Dysfunction	Assessment for bowel function
	Management of constipation
	Work-up of fecal incontinence
Cognitive Dysfunction	Assessment for cognitive deficits
	Management of cognitive deficits
Depression	Assessment for depression
	Treatment of depression
Fatigue	Assessment of fatigue
	Work-up for fatigue
	Review of medications causing fatigue
	Management of primary fatigue
Mobility/Falls	Assessment for mobility impairments
	Work-up of mobility impairments or falls
Pressure Ulcers	Assessment for risk of pressure ulcers
	Assessment for pressure ulcers in long-term facility
	Use of specialty mattresses
	Prevention of pressure ulcer
Relapses	Documentation of occurrence of relapses
	Differentiate relapse from pseudo-relapse
Sexual Dysfunction	Assessment of erectile dysfunction
	Management of erectile dysfunction
	Assessment of female sexual dysfunction
	Work-up of sexual dysfunction
	Referral to specialist with expertise in sexual problems
Spasticity	Assessment of spasticity
	Work-up of spasticity
	Management of persistent spasticity
Speech	Management of dysarthria

Swallowing	Assessment of dysphagia
	Formal tests of swallowing function
	Referral for swallowing dysfunction
	Offer of feeding tube
**General health domains of MS care**	
At Time of Diagnosis: Medical Evaluation—Appropriateness and Timeliness	Documentation of diagnostic criteria Timely initial diagnosis
At Time of Diagnosis: Patient Education	Explanation of diagnostic work-up
	Offer of information to newly diagnosed patient
Management of Exacerbations and Activities of Daily Living (ADL) Difficulties	Rehabilitation evaluation following an exacerbation Assessment of ADL difficulties
	Rehabilitation evaluation for ADL difficulties
	Treatment with steroids
	Communication of risks and benefits of steroids
	Comprehension of risks and benefits of steroids
After Diagnosis: Patient Education	Assessment for informational needs
Disease-Modifying Agents	Treatment of clinically isolated syndrome
	Disease-modifying agents for relapsing forms of MS
	Lab tests for persons on interferon beta therapy
	Lab tests for persons on high-dose interferon beta therapy
	Documentation when starting mitoxantrone or natalizumab
	Cardiac monitoring with mitoxanthrone
	Communication of risks and benefits of disease-modifying treatments
	Comprehension of risks and benefits of disease-modifying treatments
Provision of Community and Social Resources/Patient Self-Management	Assessment of problems with work or education Management of temperature
	Complementary and alternative medications
Establishment, Integration, and Coordination of Care	Visit to neurologist or physiatrist
	Access to primary care provider
	Follow-up of new medication
	Contact for usual source of care
	Documentation of consultation by referring physician
Health Promotion	Assessment of exercise habits
	Recommendation of exercise
	Assessment of general symptoms
General Preventive Care	Mammogram
	Pap smear
	Colon cancer screening
	Influenza immunization
	Pneumococcal polysaccharide vaccine
	Osteoporosis screening
Health Insurance and Disability Programs	Awareness of health insurance and disability programs

During the face-to-face discussion of indicators by Panel 2, several indicators were reworded for clarity, and a few indicators were consolidated to reduce redundancy, reducing the number of rated indicators by 2 to 86 indicators. There were 76 indicators with a final median rating of at least 7, the pre-set threshold of validity (Table 2 and Online Table 3). The remaining 10 indicators had a median rating below 7 and were excluded from further development (Online Table 4). The domains with the highest number of valid indicators include bladder dysfunction, disease-modifying agents, management of exacerbations and activities of daily living difficulties, and general preventive care (Table 3).

**Table 3.** Number of indicators by domain rated by Panel 2, and number of indicators that met thresholds for validity.

Domain Name	Number of indicators rated by Panel 2	Number of indicators that met threshold for validity
Domains of MS symptoms		
Anxiety	1	1
Bladder Dysfunction/Urinary Tract Infection (UTI)	6	6
Bowel Dysfunction	4	3
Cognitive Dysfunction	2	2
Depression	2	2
Fatigue	4	4
Mobility/Falls	2	2
Pneumonia	1	0
Pressure Ulcer	4	4
Relapses	3	2
Sexual Dysfunction	5	5
Spasticity	3	3
Speech	1	1
Swallowing	6	4
General health domains of MS care		
At Time of Diagnosis: Medical Evaluation-Appropriateness and Timeliness	2	2
At Time of Diagnosis: Patient Education	2	2
Management of Exacerbations and Activities of Daily Living Difficulties	6	6
After Diagnosis: Patient Education	1	1
Disease-Modifying Agents	9	8
Provision of Community and Social Resources/Patient Self-Management	6	3
Establishment, Integration, and Coordination of Care	6	5
Health Promotion	3	3
General Preventive Care	6	6
Health Insurance and Disability Programs	1	1
Totals	86	76

The median rating of validity by the 4 panelists with MS was within one point of the median rating of validity by the 14 panelists without MS for 76 (86%) indicators (data not shown). The ratings for two indicators were significantly different between these two groups by Wilcoxon rank-sum tests (*p* < 0.05): “Assessment of problems with work or education” was rated lower by panelists with MS versus panelists without MS (median rating of 7.5 versus 3) and “All persons with MS should be assessed for spasticity annually” was rated higher by panelists with MS versus panelists without MS (median rating of 9 versus 7).

The 76 valid measures vary in their suitability for different measurement programs (Online Table 3). There are 57 indicators that met a higher threshold of validity. Based on the literature review we concluded that 66 indicators will likely be commonly triggered among persons with MS but 10 indicators will likely be infrequently triggered. There are 19 indicators that are directly supported by results from RCTs or are endorsed by a key healthcare organization. There were 14 indicators in Online Table 3 that met the above three criteria of a higher validity threshold, commonly triggered, and are supported by either RCTs or by a key healthcare organization. Finally, based on our experience of measuring care, we concluded that 12 indicators can be obtained through administrative data but that the other 64 indicators require chart abstraction or patient surveys; of those 12 indicators that can be obtained through administrative data, six are in the domain of general preventive care, and three concern surveillance for adverse effects of disease-modifying agents.

## Discussion

Although MS presents with a wide range of symptoms, our multidisciplinary panel reached consensus on which MS symptoms were most important in each mobility stage of the disease. Such symptoms are among those known to have a strong association with health-related quality of life among persons with MS.^5^^,^^28^ Among the general health domains of MS care, the domain of disease-modifying agents was highly ranked, consistent with the large number of RCTs, meta-analyses, and guidelines that recommend their usage.^29^^,^^30^ Perhaps less predictable was that the timeliness and appropriateness of the diagnostic workup was just as highly rated. However, our interviews with persons with MS confirmed findings reported in other qualitative studies that some persons with MS still exhibited anger for being misdiagnosed for years or relief at finally being given a correct diagnosis.^31^^–^^33^ Also noteworthy are some indicators that did not meet thresholds of validity. The lowest rated indicator was antibody testing for persons using beta-interferon. Competing guidelines recommend different courses of action about this topic, reflecting uncertainty among experts.^34^^,^^35^

There is a long-standing debate within the field of health services research on the advantages and disadvantages of using patient outcomes versus medical processes of care to measure quality of care.^7^ While all stakeholders recognize that patient outcomes are extremely important, patient outcomes can be strongly associated with unmodifiable characteristics such as patient age. Therefore, to compare patient outcomes across populations, one needs to perform risk adjustment. The advantages of measuring medical care processes are that they are less likely to be sensitive to risk adjustment, and they represent an aspect of care that clinicians most directly control. However, if processes alone are used to measure quality, it may be necessary to confirm the link between performance of medical processes and improved patient outcomes.^36^^,^^37^

Measurement programs may differ in how they select indicators for implementation. Online Table 3 is provided as a sortable spreadsheet so that readers may prioritize criteria for selecting valid indicators. Programs with a small number of persons with MS should only choose indicators that are expected to be triggered frequently. Programs that use indicators for accountability purposes will prefer those that are supported by RCTs or by key healthcare organizations. Indicators measurable through administrative data are seemingly ideal, but we caution that such indicators originate from only a few domains. In addition, indicators measurable through administrative data may overestimate overall care quality because those care processes may be easier to perform. A large study of geriatric care implemented 145 quality indicators that could only be measured by reviewing the medical records, and adherence to these indicators was 55%; in the same study, 37 other quality indicators were measured using administrative data and medical record review, and the study determined that adherence to these indicators was 83% for either technique.^38^ To facilitate measurement of a comprehensive set of indicators that do not rely on administrative data, we plan to develop and pilot-test a medical chart abstraction tool and patient survey to measure care for persons with MS.

The 86 indicators presented to Panel 2 are based on a literature review and are not country-specific. Prior studies show that most indicators can be transferred to another country, but only after they are reviewed by clinicians in that country to allow for international variations in clinical practice.^39^^,^^40^

We developed a set of indicators for measuring the comprehensive care of persons with MS. The traditional application of indicators has been in health services research studies that measure whether persons are receiving appropriate care. However, in today’s healthcare environment, we envision a potentially broader use of these indicators such as certifying standards for MS centers, maintenance of board certification for healthcare providers, and application in pay-for-performance programs.

## Supplementary Material

Online Table 1

Online Table 2

Online Table 3

Online Table 4
