# Supplementary material for: Quality indicators for multiple sclerosis
Source: Mult Scler. 2010 Aug;16(8):970–80. doi: 10.1177/1352458510372394 (PMC2921149; doi:10.1177/1352458510372394)
Supplement: Online Table 1 [file msj-16-08-s001.docx]

**Online Table 1: Highly rated domains of MS symptoms, by mobility stage**

|  | Ambulatory | Ambulatory  With assistance | Wheelchair  User | Bedbound |
| --- | --- | --- | --- | --- |
| Highest tertile | **1. Depression**  **1. Fatigue**  1. Relapses  **4. Cognitive dys.**  5. Anxiety  **6. Bladder dys**  6. Weakness  8. Sexual dys. | **1. Depression**  **1. Fatigue**  1. Mobility  4. Falls/ imbalance  **5. Cognitive dys.**  **6. Bladder dys.**  7. Anxiety  **7. Spasticity/spasms** | **1. Bladder dys.**  **1. Depression**  **1. Spasticity/spasms**  **4. Cognitive dys.**  4. Mobility  6. Bowel dys.  7. Weakness  **8. Fatigue** | 1. Bowel dys.  1. Decubitus Ulcer  1. Swallowing  **4. Bladder dys.**  5. Pneumonia  6. Speech  **7. Cognitive dys.**  **7. Depression**  **7.Spasticity/spasms** |
| Middle tertile | 9. Pain/paresthesias  10. Mobility  11.Spasticity/spasms  12. Sensory loss  13. Bowel dys.  13. Sleep  13. Vision | 9. Sexual dys.  10. Weakness  11. Relapses  12. Bowel dys.  12. Pain/paresthesias  14. Sensory loss  14. Sleep  14. Tremor/ Ataxia | 9. Falls/ Imbalance  10. Sexual dys.  10. Sleep  10. Tremor/ Ataxia  13. Decubitus Ulcer  14. Pain/paresthesias  15. Anxiety  16. Vision | 10.Pain/paresthesias  10. Tremor/ Ataxia  12. Weakness  13. Vision  14. DVT  14. Sleep  16. Anxiety |
| Lowest tertile | 16. Tremor/ Ataxia  17. Falls/ imbalance  18. Vertigo  19. Headache  19. Seizures  19. Speech  19. Swallowing | 17. Vision  18. Vertigo  19. Swallowing  20. Headache  20. Seizures  20. Speech | 17. Sensory Loss  17. Swallowing  19. DVT  20. Relapses  20. Speech  22. Vertigo  23. Headache  23. Seizures | 17. Sensory loss  18. Mobility  19. Fatigue  19. Sexual dys.  21. Headache  21. Falls/Imbalance  23. Seizures  23. Vertigo |

For panel 2, the domains of Mobility and Falls/imbalance were combined to a single domain of Mobility/Falls. In addition, the domain of Weakness was covered in the other domains of Relapses, Mobility/Falls, Fatigue, and Spasticity/Spasms. Thus, the 16 domains listed in the highest tertile in this table were reduced to 14 for presentation to panel 2.

Bolded indicates that domain was rated in the highest tertile for at least three of the four mobility stages
