# Supplementary material for: Quality indicators for multiple sclerosis
Source: Mult Scler. 2010 Aug;16(8):970–80. doi: 10.1177/1352458510372394 (PMC2921149; doi:10.1177/1352458510372394)
Supplement: Online Table 2 [file msj-16-08-s002.doc]

**Online Table 2: Highly rated general health domains of MS care that apply across mobility stages of MS.**

| At time of diagnosis: Medical evaluation-appropriateness and timeliness |
| --- |
| *Disease-modifying agents |
| Establishment, integration, and coordination of care |
| At time of diagnosis: Patient education |
| *After diagnosis: Patient education |
| Management of exacerbations and activities of daily living difficulties |
| Patient self-management |
| *Health insurance and disability programs |
| Health promotion |
| General preventive care |

Domains sorted in order of the number of the panelists who rated it as indispensible to MS care

* indicates that the domain is tied with the domain directly above it.
