# Supplementary material for: Quality indicators for multiple sclerosis
Source: Mult Scler. 2010 Aug;16(8):970–80. doi: 10.1177/1352458510372394 (PMC2921149; doi:10.1177/1352458510372394)
Supplement: Online Table 4 [file msj-16-08-s004.doc]

**Online Table 4. 10 MS indicators that did not meet thresholds for validity**

| Domain | Abbreviated text of Indicator | | Full text of Indicator | |  |
| --- | --- | --- | --- | --- | --- |
| ***Bowel Dysfunction*** | |  | |  | |
|  | Use of suppositories or enemas | | IF a person with MS has constipation despite treatment with oral laxatives, THEN routine use of suppositories or enemas should be recommended if this has not previously been done. | |  |
| ***Pneumonia*** |  |  | |  | |
|  | Counseling on aspiration pneumonia risk | | IF a person has advanced MS or known swallowing problems, THEN the person should be counseled that aspiration pneumonia is a possible complication. | |  |
| ***Relapses*** |  |  | |  | |
|  | Documentation of MS subtype | | ALL persons with MS SHOULD have the subtype of their disease course documented annually. | |  |
| ***Swallowing*** |  |  | |  | |
|  | Management of persistent difficulty swallowing | | IF a person with MS has persistent difficulty swallowing, THEN evaluation of the need to adjust seating and the need for chest physiotherapy should be arranged. | |  |
|  | Evaluation of nutritional status | | IF a person with MS has swallowing difficulties for more than 1 month, THEN the person’s nutritional status should be evaluated monthly. | |  |
| ***Disease-Modifying Agents*** | | | |  | |
|  | Lab tests for neutralizing antibodies for persons on interferon beta therapy | | IF a person with MS is prescribed interferon beta therapy and there is uncertainty about its clinical effect, THEN neutralizing antibodies should be checked. | |  |
| ***Provision of Community & Social Resources/Patient Self-Management*** | | | | | |
|  | Referral to vocational rehabilitation | | IF a person with MS is experiencing difficulty with employment or education, THEN they should be referred to a specialist in vocational rehabilitation or neurorehabilitation. | |  |
|  | Assessment of self-management skills | | ALL persons with MS SHOULD be asked about their self-management skills. | |  |
|  | Assessment of coping | | All persons diagnosed with MS SHOULD be assessed for their coping styles. | |  |
| ***Establishment, Integration, and Coordination of Care*** | | | |  | |
|  | Coordinated care | | ALL persons with MS SHOULD experience coordinated care. | |  |
